# Nutrient digestibility, ruminal fermentation, and blood metabolites of growing cattle-fed fermented cassava pulp with added flavoring agents

**DOI:** 10.5455/javar.2023.j697

**Published:** 2023-09-24

**Authors:** Watcharawit Meenongyai, Kannika Wongpanit, Piyamas Phongkaew, Chunjit Kaewkunya, Theerayut Juntanam, Md. Zakirul Islam, Pichad Khejornsart

**Affiliations:** 1 Department of Agriculture and Resources, Faculty of Natural Resources and Agro-Industry, Kasetsart University Chalermphrakiat Sakon Nakhon Province Campus, Sakon Nakhon, Thailand; 2 Department of Dairy Science, Bangladesh Agricultural University, Mymensingh, Bangladesh

**Keywords:** Amyl acetate, fermented cassava pulp, flavoring agents, growing cattle, vanilla

## Abstract

**Objective:**

This study aimed to assess the effect of adding flavoring agents to fermented cassava pulp (FCPU) on nutrient utilization, ruminal fermentation characteristics, and blood metabolites in growing cattle.

**Materials and Methods:**

A duplicated 3 × 3 Latin square design was randomly assigned to six growing beef cattle. Treatments were: 1) untreated FCPU (control), 2) 0.05% *w*/*v* vanilla-flavored FCPU, and 3) 0.05% *w*/*v* amyl acetate-flavored FCPU.

**Results:**

The results showed that flavoring agents did not affect dry matter intake (DMI) or digestibility of nutrients. Rumen pH and ammonia nitrogen concentrations did not change all treatments postfeeding*.* Flavoring added to FCPU after feeding did not affect total volatile fatty acid (VFA) or VFA percentage. However, propionic acid levels tended to be lower in the vanilla-flavored FCPU group than those in the unflavored FCPU group at 0 h postfeeding (*p *< 0.01). Growing bulls fed vanilla-flavored FCPU tended to have greater fungal zoospores in the rumen than those fed amyl acetate-flavored FCPU (*p *< 0.1) at 2 h after feeding. Dietary treatments did not affect blood glucose and urea nitrogen concentrations (*p *> 0.05). However, blood triglyceride concentration was greater for cattle fed a control diet than other treatments at 0 h postfeeding (*p *< 0.05) and tended to be higher than those fed vanilla-flavored FCPU at 2 h afterfeeding (*p *< 0.1).

**Conclusion:**

It was suggested that adding vanilla or amyl acetate flavor to the FCPU showed no adverse effects on ruminal fermentation, blood metabolites, or nutritional digestibility; however, it did not increase DMI.

## Introduction 

Cattle is one of the world’s most essential livestock because meat and milk provide around 45% of human protein supply [1]. Beef cattle have three primary stages of production such as rearing, growing, and finishing. In the growing period, cattle are fed an optimal diet to increase muscle mass and maximize frame size in preparation for feedlots. However, feed accounts for 60%–65% of the cost of cattle production [2]. Therefore, it is critical to use low-cost, locally accessible industrial by-products in the ration to reduce feed costs. For instance, cassava pulp is considered a large and low-price solid by-product of the cassava flour industry [3,4], corresponding to around 10% to 15% of the initial root weight [5]. Thailand produces approximately 9.5 million tons of cassava pulp annually [6]. Fresh cassava pulp is suitable for ruminant feed since it has a high starch content, little protein, and a high fiber content. The chemical composition of cassava pulp includes 15.8%–23.4% dry matter (DM), 55.0%–74.4% nitrogen-free extract, 1.2%–2.8% crude protein (CP), 0.1%–2.4% fat, 17.9%–24.0% crude fiber, and 1.7%–2.8% ash on a DM basis [7,8]. Cassava pulp should be preserved under anaerobic conditions because of its low aerobic stability [7]. To maintain excellent DM recovery, energy, and a pleasant odor for optimal feed intake, *Lactobacillus* bacteria use water-soluble carbohydrates to create lactic acid throughout the ensiling process [9,10]. However, a mild smell of vinegar (acetic acid) or alcohol aroma could be generated in the silage and reduce feed intake [11,12]. Thus, improving the odor of fermented cassava pulp (FCPU) is one of the recommended responses to the problem.

Flavor agents are a low-cost way to increase diet palatability. The flavors are recommended in cattle feeds, including milk, vanilla, butter, orange, coconut, citric, anise, fenugreek, molasses, and maple [13]. Vanilla is the most commonly used flavoring agent in dairy cattle feed [14]. Vanilla flavor is derived from mature pods of the vanilla orchid, primarily *Vanilla planifola*, *Vanilla pompona*, or *Vanilla tahitensis* [15]. Although there are about 200 different components in vanilla, vanillin is primarily responsible for the flavor and smell [16]. Vanillin is an organic compound with the chemical formula C_8_H_8_O_3_ [17]. It is a phenolic aldehyde with the functional groups ether, hydroxyl, and aldehyde [15]. Nowadays, synthetic vanillin is utilized as a flavoring agent in food or feed more frequently than natural vanilla extract due to natural vanilla being the second most expensive spice on the market [18]. In the experiment of Harper et al. [14], the flavor did not affect concentrate intakes, but dairy cows may choose vanilla or fenugreek first when several options are available. Furthermore, amyl acetate is a flavoring agent in food and beverages [19]. It is an organic compound and an ester with the chemical formula C_7_H_14_O_2,_ and it has a scent like a fresh-fruity aroma and a sweet flavor like pear, banana, and apple [20]. Amyl acetate ester can be extracted directly from the Thai plant (Rauwenhoffia siamensis Scheff.) or synthesized through the enzymatic transesterification reaction [21]. Although amyl acetate has not previously been used as a flavoring agent in feed, Nombekela et al. [22] reported that sweet-flavored feeds can stimulate intake in early lactation cows for at least a few days. Additionally, because the volatile molecules responsible for smell spread rapidly, the scent of synthetic smells may serve as a beneficial stimulus for feed intake [14].

It is well known that adding flavoring agents can improve feed acceptance [23–26]. However, there have been limited investigations that have examined the influence of flavoring compounds added to FCPU offered to grow beef on digestion, ruminal fermentation characteristics, and blood metabolites. To determine how flavoring agents (vanilla or amyl acetate) added to fermented cassava pulp affected feed utilization, ruminal fermentation characteristics, and blood metabolite in growing beef cattle, this study aimed to add flavoring agents to fermented cassava pulp.

## Materials and Methods

### Ethical approval

Animals are managed humanely with the least number of animals, and all procedures were carried out following the Institutional Animal Care and Use Committee, Kasetsart University, Thailand (No. ACKU64-CSC-005).

### Study areas 

This study was conducted at the animal research farm of the Kasetsart University Chalermphrakiat Sakon Nakhon Province Campus in Thailand‘s northeastern region from December 2021 to March 2022. This region has a tropical *Savanna* environment with tropical monsoons for most of the year. The three seasons that occur throughout the year are winter (mid-October to mid-February), summer (mid-February to mid-May), and a rainy season (mid-May to mid-October). The temperature ranges between 22.0°C and 31.7°C, with an annual rainfall of roughly 1,645 mm and a relative humidity of 73.0% [27].

### Animal and management 

Six growing crossbred bulls (Charolais × Brahman × Thai native) average of 335 ± 51 kg of body weight (BW), and their ages average of 12 months (range: 11–13 months) were kept in separate pens with concrete flooring (3 × 4 m), with free accessed to water. Afterward, all the cattle were treated with anthelmintics (Ivermectin 1%, Vet Inter Pharma Co., Ltd., Samut Prakan, Thailand) as directed by the manufacturer to eliminate intestinal parasites and ectoparasites. Growing bulls were grouped by breed, and each breed was assigned randomly to one of the three experimental diets within its group and progressed through the three 3-week periods until exposed to all treatments.

### Experimental diets and design

The experimental design for this study was a duplicated 3 × 3 Latin square. Before the experiment, there was a 14-day adjustment period. The investigation included 63 days and was divided into three periods, including 21 days, followed by seven days of adaptation and 14 days of data collecting. The experimental diets were formulated to meet or exceed the nutritional requirements of growing beef cattle in a growing stage [28]. During the adjustment period, the cattle were offered the 18.0% CP concentrate at 1.7% BW/day, FCPU at a rate of 1.0% BW, and rice straw was fed *ad libitum*. After the 14-day adjustment period, the cattle were fed a similar concentrate at 1.7% of BW, which contained 18% CP, whereas rice straw and FCPU were fed *ad libitum*. Feed ingredients and chemical compositions of experimental diets are shown in Table 1. The FCPU was prepared by mixing it thoroughly with the flavoring agent (Vanilla or amyl acetate flavors, both from Greathill CO., Ltd., Bangkok, Thailand) at 0.05% w/v or none-added flavoring agent was used as control and then transferring it into a 200-l blue plastic drum with a closed top (Kunshan Zhida Plastic Products Co., Ltd, Jiangsu, China). The labeled ensiling drum was maintained at room temperature for 21 days before being given to the cattle. The ingredients and chemical compositions of concentrate, rice straw, and FCPU are presented in Table 1. The animals were offered twice daily at 08.00 am and 04.00 pm. Before the morning feeding, refusals were weighed and recorded daily to calculate the daily dry matter intake (DMI) [29]. To ensure a daily feed intake that provided more than 10% feed refusal [30]. The cattle always had access to water and mineral blocks.

### Data collection and laboratory analysis

The BW was measured at the beginning and end of each 14-day data collection period to determine DMI as a percent of BW. The amount of feed provided and refused was recorded daily during the 14-day collection period. Feed samples were collected from each period. During the sampling phase, rectum samples (about 500 gm) of all animals were taken every day for 3 days [31]. All fecal samples were mixed and pooled per bull, and a subsample was kept at −20°C for further analysis. Samples of feed and feces were dried at 60°C in a hot air oven to a consistent weight, then ground through a 1-mm screen (Polymix^®^ PX-MFC 90 D, Kinematica, Inc., Lucerne, Switzerland). Obtained dried samples were analyzed according to the method of AOAC [32] to determine DM, CP, ether extract (EE), and ash. The contents of neutral detergent fiber (NDF) and acid detergent fiber (ADF) were determined using an automated fiber analyzer (ANKOM 200/220, ANKOM Technology Corporation, Fairport, NY), which was adopted by the method of Van Soest et al. [33]. Acid-insoluble ash was used as an internal marker to determine the digestibility of a diet [34]. The following formula was used for calculating the apparent digestibility percentages:

**Table 1.** Feed ingredients and chemical compositions of dietary treatments (% DM).

Item	Concentrate	Rice straw	FCPU/ with unflavored	FCPU/with vanilla	FCPU/with amyl acetate
Soybean meal	13.00				
Palm kernel meal	16.00				
Lucerne meal	2.10				
Cassava chip	23.80				
Rice polishing	10.00				
Rice bran	16.70				
Used cooking oil	4.00				
Sulfur	0.10				
Dicalcium phosphate	2.20				
Salt	1.00				
Urea	2.00				
Trace mineral	1.00				
Molasses	8.00				
Total	100.00				
Chemical compositions (%DM)					
DM	84.71	91.62	15.83	15.50	15.57
CP	18.31	3.30	2.52	2.34	2.27
NDF	28.80	75.64	33.98	32.13	33.9
ADF	16.94	42.85	19.86	20.97	21.84
EE	12.40	0.73	0.4	0.42	0.39
Ash	8.91	9.68	2.13	2.25	2.19
GE (Mcal/kg)	5.15	4.74	3.97	3.90	3.99

FCPU = fermented cassava pulp, DM = dry matter, CP = crude protein, NDF = neutral detergent fiber, ADF = acid detergent fiber, EE = ether extract, GE = gross energy, SEM = standard error of the mean.

Apparent nutrient digestibility (%) = 100 × [1-(marker in feed)/(marker in feces) × (nutrient in feces)/(nutrient in feed)].

### Fermentation characteristics of the rumen

At the end of each period, rumen fluid was collected from the cattle at 0 and 2 h after morning feeding on days 14 of each data collection period using an esophageal tube and suction pump for each experimental period. A stainless-steel probe connected to an electric vacuum pump collected rumen fluid through a stomach tube. Approximately 200 ml or more of rumen fluid was disposed of without being contaminated by saliva and then was immediately measured by pH meter (Hanna Instruments, Bangkok, Thailand).

Rumen fluid samples were divided into two parts: the first part was used for volatile fatty acid (VFA) and ammonia nitrogen (NH_3_-N) analyses, and 45 ml of rumen fluid from individual cattle at each sampling period was placed into a 60 ml plastic bottle containing 5 ml of 1 M H_2_SO_4_. Then, the mixtures were centrifuged at 16,000 × gm for 15 min at 4°C, and the supernatants were collected into 25 ml test tubes, capped, and stored at −20°C until analysis [35]. The NH_3_-N content was analyzed by using automatic distillation (micro Kjedahl method) according to the method of AOAC [32]. The VFA were analyzed using high performance liquid chromatography (Agilent 1200 Series, Agilent Technologies Inc., Santa Clara, CA) equipped with a diode array detector and ZORBAX Eclipse XDB-C18 column (4.6 × 150 mm, 5 µm) (Agilent Technologies Inc., Santa Clara, CA). The carrier was 0.1 M KH_2_PO_4_, and a sample volume of 20 µl was injected. Total direct counts of bacteria, protozoa, and fungal zoospores were assessed using a hemacytometer counting chamber (BOECOTM, Hamburg, Germany) after rumen fluids were introduced to 10% saline solution at a ratio of 1:9 v/v [36].

### Serum preparation and analysis

On the last day of each period, blood samples were collected from the coccygeal vein using sampling tubes (without anticoagulant) left at room temperature for 30 min to facilitate clotting [37]. After centrifuging the serum at 3,000 × gm for 15 min, it was kept at −20°C for further analysis [38]. An auto-blood analyzer (Unicel DxC 800 Synchron Clinical System, Beckman Coulter, Inc., CA, USA) was used to examine the serum glucose, urea, and triglycerides levels.

### Statistical analysis

Statistical analysis was performed on the data obtained for the replicated 3 × 3 Latin square design using PROC MIXED of SAS [39]. The bull was considered the experimental unit, and the bull nested within the square was considered the random term. Mean values reported are least squares means. The models were as follows:

*Y_ijkl_* = *μ* + *S*_l_ + *A*_*i*(l)_ + *P_j_* + *T_k_* + *ε_ijkl_*

where *Y_ijkl _*is the variable responses, *μ* = overall mean, *S*_l _= fixed effect of square (l = 1, 2) *A_i_*_(l) _= random effect of bull nested within square (*i* = 1, 2, 3), *P_j_* = fixed effect of period (*j* = 1, 2, 3), *T_k_* = fixed effect due to treatment (*k* = 1, 2, 3), and *εijkl *= residual errors. The PDIFF option of least squares means was utilized to compare differences among treatment means. The differences in rumen fermentation, microbial population, and blood metabolite parameters between feeding times in each group were determined using a paired *t*-test. Treatment means were considered different at *p *< 0.05.

## Results and Discussion

### Dry matter intake

Table 2 shows that the addition of a flavoring agent to FCPU ingested by cattle did not affect concentrate DMI, roughage DMI, FCPU DMI (FDMI), total DMI (TDMI), and TDMI as a percentage of BW (p > 0.05). A recent study [25] found that adding vanilla flavor to calf starter increased the feed intake of male Holstein calves. Still, there was no difference in the amount consumed between the control (neutral FCPU) and any of the flavored FCPUs in this study. Studies investigating the flavor preference of ruminants by adding amyl acetate or a banana-like odor to a basal feed are scarce. However, this experiment indicated that adding the amyl acetate flavor to FCPU did not affect FDMI or TDMI. It should be demonstrated that the lactic acid flavor was a natural component of the neutral FCPU rather than an added flavor [9]. In support of this assumption, Cannas et al. [40] reported that some plant compounds can change diet preference and intake regardless of their nutritional value. A sweet taste related to water-soluble carbohydrates has increased the preference for fresh or preserved fodder in sheep and cattle [41,42]. Furthermore, the nutritional content of the feed has a strong influence on ruminant feed preferences [14], which may explain why we discovered no significant differences between the control (neutral FCPU) and the flavored FCPU since nutritional feedback would have been the same for all treatments as shown in Table 1. Previous feeding experiences influence cattle preferences through post-ingestive feedback. Additional factors, such as flavor, sight, feed texture, dam impact (through maternal ingestion, which influences newborn feeding), and their interactions may also play a role in ruminant flavor preferences [43]. Launchbaugh and Provenza [44] reported that odor is only essential in feed selection when it is accompanied by taste. In addition, animals choose individual feed items using their senses of smell, sight, and taste [45]. Our findings could be comparable to Carlotto et al. [26] showed no significant differences between the animal groups for concentrate DMI, hay DMI, milk DMI, and total DMI when milk or citric flavor agents were added to the diets of young dairy calves. Nedelkov et al. [43] found that the synthetic flavors used in the diets did not affect weaned lambs and calves’ feed consumption.

**Table 2.** DM and nutrient intake of growing bull fed FCPU with differing flavoring agents.

Item	FCPU (Control)	FCPU+ Vanilla	FCPU+ Amyl acetate	SEM	*p*-value
Concentrate intake (kg/day)	6.72	7.09	6.98	0.46	0.60
Roughage intake (kg/day)	1.92	1.80	1.94	0.15	0.92
FCPU intake (kg/day)	5.08	4.12	4.28	0.46	0.40
Total DM intake (kg/day)	13.50	13.01	13.15	0.94	0.81
DM intake (% of BW)	3.21	3.14	3.14	0.06	0.73

FCPU = fermented cassava pulp, SEM = standard error of the mean.

### Nutrient digestibility

In this study, adding flavoring agents to FCPU did not affect the digestibility of DM, organic matter (OM), CP, EE, NDF, or ADF, as shown in Table 3. A similar feed intake between the bulls fed flavored FCPU in our study (Table 2) may have resulted in a similar passage rate of the digester through the gastrointestinal tract [46], explaining why there was no significant difference between the treatments. Nutrient digestibility in ruminants is influenced by the feed‘s ability to be broken down by rumen bacteria, small intestine enzymatic digestion, and passing rate [47]. Although we have not found previous studies on adding amyl acetate to ruminant diets, our results found that it did not affect nutrient digestibility. This finding suggests that amyl acetate does not interfere with microbial activity in the rumen [48] and results in similar nutrient digestibility. Furthermore, it is well known that vanillin is a primary component of vanilla [16]. Although vanillin has been previously reported to inhibit mixed population digestion in rumen fluid [49], which may affect nutrient metabolism, no effect of vanillin on nutrient digestibility was observed in this study. This finding could be comparable to Borneman et al. [49] demonstrated that the phenolic compound (vanillin) did not affect the *in vitro* DM digestibility of cellulose. Piran Filho et al. [23] also reported that feeding aromatic compounds such as licorice, caraway, cinnamon, and vanilla to Angus Nellore cross bulls did not affect the apparent total tract digestibility parameters. Furthermore, Karimov et al. [50] demonstrate that vanillin combined with probiotic strains (*Bifidobacterium longum* and *Bifidobacterium adolescentis*/*Lactobacillus acidophilus*) increases wheat digestibility, possibly due to changes in the rumen microbiome.

### Ruminal fermentation

In Table 4, the flavorings applied to FCPU on ruminal fermentation are shown. Ruminal pH at all intervals postfeeding showed no significant difference between treatments (*p *> 0.05). The ruminal pH ranged from 7.03 to 7.05 at 0 h postfeeding, then dropped to 6.55 to 6.68 at 2 h postfeeding. The ruminal pH for both the vanilla-flavored FCPU treatment and the unflavored FCPU treatments significantly dropped at 2 h postfeeding (*p *< 0.05). The NH_3_-N content in the rumen varied between 7.68 and 10.00 mg/dl at 0 h after feeding and increased to 13.85–13.96 mg/dl at 2 h after feeding, with no significant differences seen between treatments. However, all treatments slighty increased 2 h after feeding (*p *< 0.05). At 0 h postfeeding, the molar proportions of acetic acid (C2) and butyric acid (C4) in the rumen of growing bulls were not significantly different among treatments (*p *> 0.05). The proportions of propionic acid (C3) in the rumen of growing bulls fed vanilla-flavored FCPU tended to be lower than in the control group (*p *= 0.09), leading to an increase in the C2:C3 ratio (*p *< 0.1) compared to those fed unflavored FCPU (control). However, compared to the control, there was a significant increase (39.7%) after feeding for 2 h. The concentrations of total, volatile fatty acid (TVFA) in the rumen of growing bulls fed vanilla-flavored FCPU tended to be lower than those fed amyl acetate-flavored FCPU (*p *= 0.09). However, there was no significant difference in the molar proportions of C2, C3, C4, TVFA content, or the C2:C3 ratio at 2 h postfeeding (*p *> 0.05). The molar proportion of C2 tended to decrease after 2 h postfeeding compared to 0 h (*p *= 0.06), leading to an increasing trend in the C2:C3 ratio (*p *= 0.08). The TVFA at 2 h postfeeding was significantly higher (*p *< 0.05) for the control and vanilla-flavored FCPU feeding groups compared to 0 h postfeeding.

**Table 3.** Effect of adding flavoring agents in FCPU on nutrients digestibility coefficient of growing bull (%).

Item	FCPU (Control)	FCPU+ Vanilla	FCPU+ Amyl acetate	SEM	*p*-value
DM	70.75	68.90	69.51	1.64	0.87
OM	74.09	71.28	70.73	1.44	0.18
CP	62.77	65.80	64.80	1.83	0.83
EE	91.33	92.42	88.80	1.06	0.38
NDF	47.13	48.54	48.57	2.49	0.12
ADF	40.62	43.24	44.28	2.81	0.22

FCPU = fermented cassava pulp, SEM = standard error of the mean.

Although the pH of *in vitro* rumen mixed cultures has been reported to increase [51] and decrease [52] at different concentrations of vanillin, the rumen pH was not affected by any flavored FCPU (*p* > 0.05) in this study. This is possibly because vanillin did not modify rumen fermentation, as shown in Table 4, where the molar proportion and TVFA concentration were similar for all treatments at 2 h postfeeding. Goto et al. [53] reported that the molar proportion of VFA and the accumulation of VFA in the rumen is responsible for a lower rumen pH. Furthermore, Vargas et al. [54] reported that the ruminal pH decreased due to the fermentation of carbohydrates and VFA accumulation in the rumen, which explained why the ruminal pH significantly decreased after 2 h postfeeding.

Protein is broken down into NH_3_-N, absorbed, converted to urea in the liver, and excreted in the urine when ruminal degradable protein levels exceed those needed for ruminal bacteria [55]. Essential oil mixes (thymol, eugenol, vanillin, and limonene) have been shown to suppress ammonia-producing bacteria in the rumen and reduce amino acid deamination [56]. *In vitro* studies have also found that essential oil mixes inhibit the activity of ammonia-producing bacteria, while other bacteria can adapt to them [57]. In an *in vivo* trial, essential oil supplementation reduced the number of ammonia-producing bacteria in sheep fed a low-protein diet [58], whereas essential oil was ineffective in the same experiment when fed a high-protein diet, indicating that the inhibitory impact of essential oil on ammonia-producing bacteria may be diet-dependent. The rise in ammonia concentration at 2 h post-feeding (*p* < 0.05) is additional evidence that the flavoring agents in our experiment had no negative effects on the rumen bacteria population (Table 5). In recent studies [51,52], NH_3_-N content was reduced when vanillin was added to the culture medium, but it was not impacted by any flavored FCPU in the present study (*p* > 0.05). This result demonstrated that flavor agents did not affect protein breakdown or amino acid fermentation in the rumen [52]. This was further substantiated by the similar concentration of blood urea nitrogen (Table 6), which primarily arises from the deamination of amino acids [59].

There are no other reports in the literature on the effects of amyl acetate on rumen fermentation. This study suggests that adding amyl acetate to FCPU had no negative effect on rumen fermentation. Vanillin at the maximum dose (5 gm/l) was observed to slightly decrease the amount of C2 (−2.0%), while doses of 0.5 gm/l did not affect the overall rumen fermentation [51]. In our study, flavoring agents were used at a dose of 0.05% *w*/*v*. This may be an appropriate dose, which may not have modified rumen microbial fermentation. In a previous study [52], vanillin showed decreased total VFA contents, which resulted from the lower degradability of the feedstuff. The similarity in apparent digestibility (Table 3) could explain why we did not find a difference in the concentration of TVFA between the treatments. This result is comparable to Vargas et al. [54], who reported that greater production of VFA was found when diets had higher digestibility. The molar proportion of C2 tended to decline after 2 h postfeeding, compared to 0 h, suggesting that the antimicrobial effects of vanillin on cellulolytic bacteria may be responsible for this result, which led to a decrease in the amount of acetate. According to Varel and Jung [60], who reported that vanillin may interfere with the attachment of *Bacteroides succinogenes* to cellulose, leading to decreased cellulose degradation. On the other hand, vanillin had no effects on rumen microbial populations such as *Ruminococcus albus*, *Fibrobacter succinogenes*, *Selenomonas ruminantium*, *Megasphaera elsdenii*, and *Streptococcus bovis *[52]. Thus, the efficacy of vanillin was mixed in animal studies.

### Microbial population

The effects of supplementing flavoring agents in FCPU on the microbial population are shown in Table 5. The population of bacteria, protozoa, and fungal zoospore in the rumen at 0 h postfeeding was not significantly different between the treatments (*p *> 0.05). Furthermore, 2 h postfeeding, the populations of bacteria and protozoa were also not statistically different between the treatments (*p *> 0.05). Although vanillin has been shown to exhibit potent antibacterial and antifungal activity in previous studies [61,62], surprisingly, vanilla-flavored FCPU did not affect the populations of bacteria and protozoa in this study when compared with the control, whereas growing bulls fed vanilla-flavored FCPU tended to have greater fungal zoospores than those fed amyl acetate-flavored FCPU (*p *= 0.09), as shown in Table 5. The microbial populations at 2 h post-feeding compared to 0 h post-feeding did not different (*p* > 0.05), except for bacteria in the unflavored FCPU feeding group (*p* = 0.06) and fungal zoospores in the amyl acetate-flavored FCPU feeding group (*p* = 0.06) that found an increasing trend. In contrast, protozoa found a decreasing trend in the rumen of the vanilla-flavored FCPU feeding group (*p* = 0.06). An appropriate amount of flavoring agents might be part of the explanation for the microbial population being unaffected by the antimicrobial properties of vanillin [51]. The result agrees with research by Benchaar et al. [63], which showed that supplementing dairy cow diets with an essential oil blend comprising thymol, eugenol, vanillin, and limonene had no impact on the total viable bacteria, cellulolytic bacteria, or protozoa in the rumen. The ammonia synthesis due to the effect of vanillin may be partially attributable to the decline in protozoan populations. Vanillin may be partially responsible for the decrease in NH_3_-N in the rumen due to the declining protozoan numbers associated with higher vanillin dosages [52].

**Table 4.** Effect of adding flavoring agents in FCPU on ruminal pH, NH_3_-N, and VFA production of growing cattle.

Item	FCPU (Control)	FCPU+ Vanilla	FCPU+ Amyl acetate	SEM	*p*-value^1^
pH					
0 h	7.03^A^	7.05^A^	7.05	0.06	0.99
2 h	6.55^B^	6.57^B^	6.68	0.07	0.64
*p*-value^2^	<0.01	0.02	0.10		
NH_3_-N (mg/dl)					
0 h	7.68^B^	8.56^B^	10.00^B^	0.51	0.14
2 h	13.85^A^	13.96^A^	13.85^A^	0.73	1.00
*p*-value	0.01	<0.01	0.04		
Acetic acid (C2, mol/100 mol)					
0 h	52.33	60.42	56.08	2.71	0.35
2 h	54.18	54.86	54.84	2.99	0.99
*p*-value	0.57	0.06	0.69		
Propionic acid (C3, mol/100 mol)					
0 h	23.26	14.65	18.49	1.69	0.09
2 h	21.96	20.47	20.85	1.97	0.96
*p*-value	0.60	0.10	0.47		
Butyric acid (C4, mol/100 mol)					
0 h	24.42	24.94	25.43	1.84	0.95
2 h	23.86	24.67	24.31	2.08	0.96
*p*-value	0.76	0.94	0.57		
Total VFA (mmol/l)					
0 h	76.11^B^	69.23^B^	95.38	5.48	0.09
2 h	96.04^A^	93.38^A^	96.84	4.39	0.97
*p*-value	0.02	0.01	0.62		
C2:C3 ratio					
0 h	2.57	4.52	3.40	0.37	0.06
2 h	3.03	3.08	3.07	0.35	1.00
*p*-value	0.45	0.08	0.64		

^A, B^Means with different superscript within the same column are statistically significant differences (*p* < 0.05). ^1^Treatment effect,^2^ Feeding time effect.

FCPU = fermented cassava pulp, SEM = standard error of the mean.

### Blood metabolites

The blood glucose and urea-N levels shown in Table 6 were unaffected by dietary treatments (*p *> 0.05). This suggests similarities in the metabolism of carbohydrates and proteins between the growing bulls fed FCPU flavored with vanilla and amyl acetate and the control group. However, serum triglyceride concentration was higher for the control group than other treatments at 0 h postfeeding (21.50, 17.17, and 17.00 mg/dl, respectively, *p* < 0.05). Also, it tended to be greater than those fed a vanilla-added treatment at 2 h postfeeding (23.67, 17.50, and 20.67 mg/dl, respectively, *p *= 0.07). The effects of flavoring agents on blood metabolites in growing cattle have not been widely studied. Serum glucose concentrations were increased at 2 h postfeeding (*p *< 0.01) compared to 0 h postfeeding in the amyl acetate-flavored FCPU feeding group. In contrast, in the unflavored FCPU feeding group, there was an increasing trend (*p *= 0.08). Serum urea-N levels for all treatments were significantly increased after 2 h postfeeding compared to 0 h postfeeding (*p *< 0.05). In contrast, serum triglyceride levels did not differ between the feeding times (*p *> 0.05). Blood metabolites were used as indicators of nutrient availability for nutrient utilization [64]. After feeding, the blood glucose level increases due to the breakdown of carbohydrates. The serum glucose concentration of growing bulls fed vanilla-flavored FCPU did not rise at 2 h postfeeding. We suspect glucose may be converted to lactate or other metabolites in the brush border membrane of small intestine cells. Vanillin supplementation has been shown to reduce the levels of some blood metabolites, including glucose, in prior studies [65]. However, adding vanilla or amyl acetate to the FCPU of growing bulls resulted in no changes in values of serum glucose or urea-N in this study. This is consistent with the findings of Fontoura et al. [66], who observed that dietary organic acid and pure botanical (thymol and vanillin) supplementation did not substantially impact blood glucose, urea-N, or triglyceride concentrations in Holstein dairy cows. Excessive ruminal NH_3_-N is absorbed through the rumen wall into the portal blood, where most of it is transformed into urea [67]. As a result, ammonia utilized from the rumen is used to make urea in the liver, and the amount of urea in the blood is significantly associated with the amount of NH_3_-N in the rumen [68]. No changes in serum urea-N concentration were expected because the ruminal concentration of NH_3_-N was unaffected by flavoring agent supplementation in our investigation. As per the blood metabolites of growing bulls, adding vanilla flavor to FCPU tended to reduce the concentrations of serum triglycerides in growing bulls compared to the control group. This suggests that growing bulls fed vanilla-flavored FCPU results in increased insulin sensitivity and metabolism of triglycerides [69,70]. According to a previous study [71], using triglycerides as an energy source led to decreased serum triglyceride concentrations in crossbred dairy calves. Additionally, other research [72–74] shows vanillin significantly lowers blood triglyceride levels in rats with hyperlipidemia induced by a high-fat diet.

**Table 5.** Effect of adding flavoring agents in FCPU on total viable count of bacteria, protozoal and anaerobic fungal zoospore in growing cattle.

Item	FCPU (Control)	FCPU+ Vanilla	FCPU+ Amyl acetate	SEM	*p*-value^1^
Bacteria, ×10^9^cell/ml					
0 h	1.75	1.42	1.91	0.35	0.80
2 h	5.08	3.24	3.44	0.78	0.63
*p*-value^2^	0.06	0.30	0.10		
Protozoa, ×10^5^ cell/ml					
0 h	6.95	3.14	9.17	1.90	0.45
2 h	3.18	2.03	5.12	0.92	0.44
*p*-value^2^	0.15	0.06	0.16		
Fungal zoospore, ×10^6^ cell/ml					
0 h	1.71	1.43	0.81	0.27	0.13
2 h	1.54	2.34	0.90	0.26	0.09
*p*-value^2^	0.48	0.27	0.06		

^1^ Treatment effect,^2^ Feeding time effect.

FCPU = fermented cassava pulp, SEM = standard error of the mean.

**Table 6.** Blood profile of growing cattle fed FCPU with differing flavoring agents.

Item	FCPU (Control)	FCPU+ Vanilla	FCPU+ Amyl acetate	SEM	*p*-Value^1^
Glucose, mg/dl					
0 h	86.50	93.83	88.83	3.26	0.65
2 h	101.50	90.33	97.17	3.26	0.10
*p*-value^2^	0.08	0.25	<0.01		
Urea-N, mg/dl					
0 h	8.83^B^	8.50^B^	9.00^B^	0.65	0.87
2 h	10.83^A^	10.67^A^	11.17^A^	0.66	0.88
*p*-value^2^	0.02	0.02	<0.01		
Triglyceride, mg/dl					
0 h	21.50^a^	17.17^b^	17.00^b^	1.22	0.04
2 h	23.67	17.50	20.67	1.73	0.07
*p*-value^2^	0.15	0.32	0.11		

^a,b^ Means with different superscript within the same row are statistically significant differences (*p* < 0.05).

^A, B ^Means with different superscript within the same column are statistically significant differences (*p* < 0.05).

^1^ Treatment effect,^2^ Feeding time effect, FCPU = fermented cassava pulp, SEM = standard error of the mean.

## Conclusion

The results of this study suggest that neither vanilla nor amyl acetate improved the DMI of FCPU or affected the nutrient digestibility of growing bulls. Amyl acetate flavor did not affect rumen fermentation, while vanilla flavor had a slight effect on rumen fermentation by tending to reduce the C3 proportion. Vanilla or amyl acetate flavors did not affect the microbial population, while vanilla flavor decreased the concentration of serum triglycerides. Although adding amyl acetate or vanilla flavors to FCPU did not potentially improve DMI in growing bulls, our findings suggest that supplementation at 0.05% *w*/*v* did not affect nutrient digestibility, rumen fermentation, or blood metabolites. However, as FCPU still has a strong odor, flavoring agent addition in concentration based on local feed supplies was possible in the future.

## List of abbreviations

ADF, acid detergent fiber; BW, body weight; C2, acetate; C3, propionate; C4, butyrate; CP, crude protein; DM, dry matter; DMI, dry matter intake; EE, ether extract; FCPU, fermented cassava pulp; GE, gross energy; NDF, neutral detergent fiber; NH_3_-N, ammonia nitrogen; SEM, standard error of the mean; TDMI, total dry matter intake; TVFA, total, volatile fatty acid; urea-N, urea nitrogen; VFA, volatile fatty acid; w/v, weight/volume.
